# The trend of geriatric health research and the challenge of health system translation in Nigeria

**DOI:** 10.1186/1472-6963-14-S2-P3

**Published:** 2014-07-07

**Authors:** Musa Abubakar Kana

**Affiliations:** 1 Department of Community Medicine, Faculty of Medicine, Kaduna State University, Kaduna, Kaduna State, Nigeria

## Background

Nigeria is expected to be the nation with the 11th highest population of older persons by 2015. This demographic transition will have implications for the medical care services. Currently, there is no geriatric social health insurance policy nor comprehensive geriatric healthcare services in the country. The minimal health system response may be due to lack of comprehensive knowledge of geriatric disease burden, pattern and healthcare needs. This paper aims to describe the trend and pattern of geriatric health research publications, and to review the current geriatric health policies in Nigeria.

## Materials and methods

A systematic review of published reports in PubMed was performed for the period of January 1990 to April 2014. The review of current geriatric health policies was conducted by document analyses of an annotated national bibliography on digitized health policies and guidelines.

## Results

A total of 38 policies and guidelines of the Federal Ministry of Health were reviewed and none of them targeted geriatric health. The systematic review identified 52 eligible studies out of 3519 studies. Twenty-five studies (48%) were community-based studies, twenty (38%) were conducted in hospital settings, while only one was carried out in an old people’s home and four were narrative literature reviews. Twelve studies (23%) were done in a rural area, 24 (46%) in an urban area and 11 (21%) report findings from both locations.

One study reported the health of the elderly and economic policies (1), elderly destitution (1), health implication of ageing (1), attitude to ageing (2), care for the elderly (3), quality of life and life satisfaction (3), nutrition (2) and physical activity (1). The morbidity pattern reported from the reviewed articles were as follows: medical morbidities (8), geriatric emergencies and admissions (2), dental problems (5), surgical morbidities (1), nosocomial infection (1), mental health morbidities (6), orthopaedic (2), otorhinolaryngology (3), urology (1) and visual morbidities (5).

## Conclusions

The findings underscore a geriatric health research and policy needs. It is recommended that resources be invested into collaborative research for the development and implementation of evidence based geriatric health policies.

